# Tristetraprolin Overexpression in Non-hematopoietic Cells Protects Against Acute Lung Injury in Mice

**DOI:** 10.3389/fimmu.2020.02164

**Published:** 2020-09-02

**Authors:** Ishita Choudhary, Thao Vo, Chandra S. Bathula, Richa Lamichhane, Brandon W. Lewis, Jayme Looper, Samithamby Jeyaseelan, Perry J. Blackshear, Yogesh Saini, Sonika Patial

**Affiliations:** ^1^Department of Comparative Biomedical Sciences, School of Veterinary Medicine, Louisiana State University, Baton Rouge, LA, United States; ^2^Veterinary Clinical Sciences, School of Veterinary Medicine, Louisiana State University, Baton Rouge, LA, United States; ^3^Pathobiological Sciences, School of Veterinary Medicine, Louisiana State University, Baton Rouge, LA, United States; ^4^Signal Transduction Laboratory, National Institute of Environmental Health Sciences, Research Triangle Park, NC, United States

**Keywords:** tristetraprolin, *Zfp36*, acute lung injury, inflammation, neutrophil

## Abstract

Tristetraprolin (TTP) is a mRNA binding protein that binds to adenylate-uridylate-rich elements within the 3′ untranslated regions of certain transcripts, such as tumor necrosis factor (*Tnf*) mRNA, and increases their rate of decay. Modulation of TTP expression is implicated in inflammation; however, its role in acute lung inflammation remains unknown. Accordingly, we tested the role of TTP in lipopolysaccharide (LPS)-induced acute lung injury (ALI) in mice. LPS-challenged TTP-knockout (TTP^KO^) mice, as well as myeloid cell-specific TTP-deficient (TTP^myeKO^) mice, exhibited significant increases in lung injury, although these responses were more robust in the TTP^KO^. Mice with systemic overexpression of TTP (TTP^ΔARE^) were protected from ALI, as indicated by significantly reduced neutrophilic infiltration, reduced levels of neutrophil chemoattractants, and histological parameters of ALI. Interestingly, while irradiated wild-type (WT) mice reconstituted with TTP^KO^ hematopoietic progenitor cells (HPCs) showed exaggerated ALI, their reconstitution with the TTP^ΔARE^ HPCs mitigated ALI. The reconstitution of irradiated TTP^ΔARE^ mice with HPCs from either WT or TTP^ΔARE^ donors conferred significant protection against ALI. In contrast, irradiated TTP^ΔARE^ mice reconstituted with TTP^KO^ HPCs had exaggerated ALI, but the response was milder as compared to WT recipients that received TTP^KO^ HPCs. Finally, the reconstitution of irradiated TTP^KO^ recipient mice with TTP^ΔARE^ HPCs did not confer any protection to the TTP^KO^ mice. These data together suggest that non-HPCs-specific overexpression of TTP within the lungs protects against ALI via downregulation of neutrophil chemoattractants and reduction in neutrophilic infiltration.

## Introduction

Acute lung injury (ALI) and its severe form, acute respiratory distress syndrome (ARDS), are serious health concerns due to a high rate of mortality (1). ALI is characterized by elevated levels of proinflammatory mediators, exaggerated neutrophil recruitment, and compromised pulmonary epithelial-endothelial barrier, resulting in increased vascular permeability (2). Non-cardiogenic pulmonary edema, characterized by excessive accumulation of protein-rich edematous fluid and inflammatory cells in the alveolar spaces, results in hypoxemia in ARDS that requires aggressive clinical management including mechanical ventilation (1). Despite significant health burdens posed by these diseases, the identity of key cellular and molecular players of host defense against ALI remains unclear.

Zinc finger protein 36 (ZFP36), commonly known as tristetraprolin (TTP), is an mRNA binding protein that binds to adenylate-uridylate-rich elements (AREs) within the 3′ untranslated regions (3′UTRs) of target mRNAs and increases their rate of decay (3). Germline TTP-knockout (TTP^KO^) mice exhibit the spontaneous development of a systemic inflammatory syndrome characterized by cachexia, erosive arthritis, myeloid hyperplasia, dermatitis, conjunctivitis, and autoimmunity (4). These phenotypes were shown to be essentially completely prevented in TTP^KO^ mice with either TNF receptor deficiency, or when TTP^KO^ mice were treated with anti-TNF antibodies (5). Biochemical studies demonstrated that TTP binds to AREs within the tumor necrosis factor (*Tnf*) mRNA 3′UTR and results in *Tnf* mRNA degradation under normal conditions (6, 7). Subsequent reports have shown that a number of other pro-inflammatory mediators including CXCL1 (8, 9), CXCL2 (8), IL-10 (10), IL-17 (11), CCL3 (12), and IL-23 (13) are also regulated by TTP (14). Recently, using a systemic TTP overexpression (TTP^ΔARE^) mouse model, we demonstrated protective effects of enhanced TTP levels in chronic immune-mediated inflammatory diseases including mouse models of arthritis, psoriasis, and autoimmune encephalomyelitis (15). TTP^ΔARE^ mice lack AREs in the 3′UTR of the endogenous TTP gene (*Zfp36*) that results in increased stability of TTP mRNA and, in turn, moderately increased expression of TTP protein in essentially all the tissues (15). Together, these studies have indicated that TTP may be an endogenous anti-inflammatory protein and that enhancing its levels may be beneficial against various chronic inflammatory diseases.

In the present study, we investigated the role of TTP in regulating lung inflammation in a mouse model of ALI. Using an oropharyngeal aspiration approach, lipopolysaccharide (LPS)-induced ALI was modeled in adult mice, and the animals were monitored for signs of ALI. To identify the protective role of TTP in a cell-specific manner, we performed bone marrow irradiation and reconstitution experiments in wild-type (WT), TTP^KO^ (4), and systemic TTP overexpression (TTP^ΔARE^) mice (15). Our findings elucidate cell-specific roles of TTP in protection against ALI, and indicate that TTP is an important modulator of endotoxin-induced ALI.

## Materials and Methods

### Mice

*Zfp36* Floxed mice (*Zfp36*^Flox/Flox^) (16) were crossed with LysMcre recombinase expressing mice (17) to generate mice for experimental (Cre^+/+^/*Zfp36*^Flox/Flox^; TTP^myeKO^) and control (Cre^–/–^/*Zfp36*^Flox/Flox^; Cre^+/+^/*Zfp36*^WT/WT^) groups. Genotype status of progeny was determined by polymerase chain reaction (PCR) as described previously (16). TTP knockout mice (TTP^KO^) and TTP overexpression mice (TTP^ΔARE^) have been described before (4, 15). All the animal experiments were performed in accordance with principles and procedures outlined in the National Institute of Health Guide for the Care and Use of Laboratory Animals and were approved by the Louisiana State University Animal Care and Use Committee.

### LPS Challenge

Both male and female adult (8–10-week-old) mice were used for experiments. Mice were anesthetized with isoflurane/oxygen followed by administration of 10 μg Lipopolysaccharide (LPS) from *Escherichia coli* O111:B4 (L4391, Sigma-Aldrich) per mouse dissolved in sterile endotoxin-free saline (50 μl total volume), or an equivalent volume of sterile endotoxin-free saline as a vehicle control, via oropharyngeal aspiration (18). Mice were observed for signs of distress including anorexia, weight loss, hunched posture, ruffled haircoat, labored breathing, and dehydration every 8–12 h post LPS challenge. Mice exhibiting at least four of these clinical signs were humanely euthanized before the end of the study.

### LPS-Induced Acute Lung Injury

Following saline or LPS treatment, mice were anesthetized with 2,2,2-tribromoethanol (Sigma-Aldrich, St. Louis, MO, United States) at the indicated time points, and mid-line laparotomy was performed. Briefly, bronchoalveolar lavage fluid (BALF) was harvested from the right lung. Recovered BALF was processed and analyzed for total and differential cell counts by routine methods (19). Unlavaged left lung lobes were fixed in 10% neutral buffered formalin (NBF) and used for preparation of slides for histopathological evaluation. Right lung lobes were snap-frozen and stored at −80°C.

### Measurement of Cells in BALF

Bronchoalveolar lavage fluid was harvested and centrifuged at 500 × *g* for 5 min, and the supernatant was stored at −80°C for further analyses. The cell pellet was resuspended in 500 μl of PBS and total cell counts were determined using a hemocytometer (Brightline, Horsham, PA, United States). Cytospins were prepared using 200 μl of cell suspension (Statspin Cytofuge 2; HemoCue, Brea, CA, United States) followed by differential staining (Modified Giemsa kit; Newcomer Supply, Middleton, WI, United States).

### Measurement of Cytokines in BAL

Mouse cytokine and chemokine levels were assayed in cell-free BALF supernatant using Luminex-XMAP–based assay (MCYTOMAG-70K), according to the manufacturer’s instructions (EMD Millipore, Billerica, MA, United States).

### Histology

Five micrometer sections of lung were stained with Hematoxylin and Eosin (H&E) for routine histology. *Histology*: A semiquantitative histopathological scoring system was used to analyze the sections as follows: (1) Consolidation (percent of total surface area of lung section affected); (2) bronchiolitis (0, no bronchioles affected; 1, one bronchiole affected; 2, between 2–4 bronchioles affected; 3, more than 4 bronchioles affected); (3) perivascular edema (1, minimal; 2, mild; 3, moderate; 4, severe); (4) perivascular inflammation/inflammatory cells (0, absent; 1, minimal; 2, mild; 3, moderate; 4, severe); (5) airspace edema (1, minimal; 2, mild; 3, moderate; 4, severe); (6) airspace hemorrhages (0, absent, 1, patchy, mild; 2, extensive, moderate; 3, extensive, severe). Slides were graded in a blinded manner without knowledge of sex and treatment groups.

### Bone Marrow Transplantation

Bone marrow transplantation experiments were performed as described previously (20). Briefly, 8–10-week old recipient mice were irradiated with 6 Megavolt X-rays from a Linear Accelerator (Varian Clinac 21EX) with two (dorsal and ventral) 525-rad (525 cGy) doses. To prepare bone marrow cells for transplantation, femur bones of donor mice were flushed to collect bone marrows, and single cell suspensions were prepared. A total of 8 × 10^6^ cells were injected into the tail vein of lethally irradiated recipient mice. Reconstituted recipient mice were given 0.2% neomycin sulfate dissolved in acidified water for the first 2 weeks post-transplantation. LPS-challenge experiments were performed 8 weeks post bone marrow reconstitution, which has been previously shown to be an optimal period for repopulation of resident alveolar macrophages with donor cells following total body irradiation (21).

### Immunoblotting

Lung tissue was lysed using Pierce^TM^ RIPA buffer (Thermo Fisher Scientific, Waltham, MA, United States) supplemented with Pierce^TM^ protease inhibitor mixture (Thermo Fisher Scientific, Waltham, MA, United States) and phosphatase inhibitors (10 mM sodium fluoride and 1 mM sodium orthovanadate). Tissues were mechanically homogenized using a bead beater (Thermo Fisher Scientific, Waltham, MA, United States). Tissue lysates were centrifuged (13,000 × *g*, 10 min, 4°C) to remove insoluble material and protein concentration of the supernatants was measured through Bradford assay (Bio-Rad Laboratories, Hercules, CA, United States). Equivalent amounts of denatured protein was separated on a 4–12% Bis-Tris plus precast gels (Invitrogen, Carlsbad, CA, United States), transferred on to PVDF membrane (Invitrogen, Carlsbad, CA, United States) and probed with a 1:5000 dilution of rabbit antiserum raised against a recombinant mouse TTP-maltose binding protein fusion (15) followed by incubation with horseradish peroxidase-conjugated goat anti-rabbit IgG (Bio-Rad). Signal was determined using SuperSignal West Pico chemiluminescent substrate (Pierce) on X-ray film.

### Statistical Analysis

Significant differences among groups were determined by one-way analysis of variance (ANOVA) followed by Tukey’s *post hoc* test for multiple comparisons except for cytokine assays where two-way ANOVA was used. Measurements from two groups were compared using Student’s *t*-test assuming unequal variance. All data were expressed as mean ± SEM. A *p-*value < 0.05 was considered statistically significant. Statistical analyses were performed using GraphPad Prism 7.0 (GraphPad Software, La Jolla, CA, United States).

## Results

### Germline Deletion of TTP Increases the Severity of LPS-Induced ALI in Mice

**FIGURE 1:**
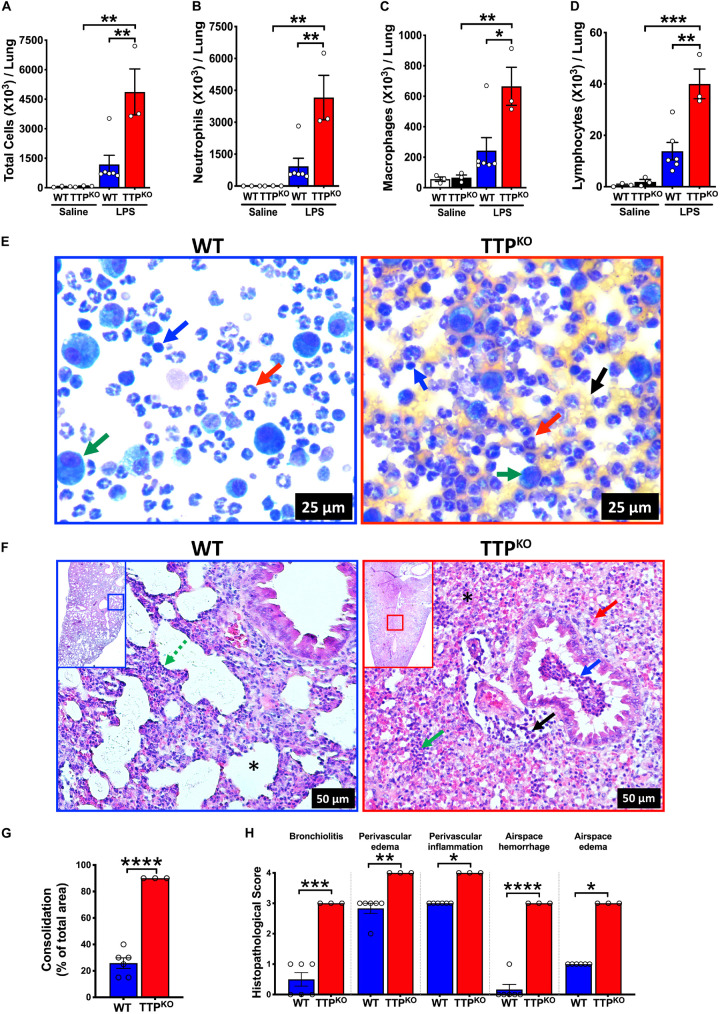
Germline deletion of TTP increases the severity of LPS-induced ALI in mice. Total cell counts **(A)** in the BALF from adult saline administered and LPS-challenged WT and TTP^KO^ mice. Differential cell counts are shown for neutrophils **(B)**, macrophages **(C)**, and lymphocytes **(D)**. Data are represented as mean ± SEM. Statistical analysis was performed by one-way ANOVA followed by Tukey’s correction for multiple comparison test. *p < 0.05; **p < 0.01; ***p < 0.001. *N* = 3 each for WT and TTP^KO^ saline controls; *N* = 6, WT group; *N* = 3, TTP^KO^ group for LPS-challenge group. Three LPS-challenged TTP^KO^ mice succumbed to LPS-challenge before 72 h and were not lavaged for further analyses. Representative photomicrographs of Wright–Giemsa stained BALF cytospins of LPS-challenged WT (**E**; left panel) and LPS-challenged TTP^KO^ (**E**; right panel) mice. Neutrophil (red arrow), macrophage (green arrow), lymphocyte (blue arrow), red blood cell (black arrow) (original magnification ×400). Representative photomicrographs **(F)** from H&E-stained left lung lobe sections from adult LPS-challenged WT (**F**; left panel) and LPS-challenged TTP^KO^ (**F**; right panel) mice. Septal thickening (green broken arrow), intra-alveolar neutrophilic infiltrates (green arrow), intra-alveolar red blood cells (red arrow), bronchiolar lumen neutrophilic accumulation (blue arrow), and perivascular cellular infiltration (black arrow). Asterisk represents alveolar space that is minimally affected (**F**; Left) or severely consolidated with blood and neutrophils (**F**; right) (original magnification ×200). Semiquantitative histopathological scoring for consolidation **(G)** is shown as a percent of total surface area of the lung section affected in LPS-challenged WT and LPS-challenged TTP^KO^ mice. Semiquantitative histopathological scoring of lung sections for bronchiolitis, perivascular edema, perivascular inflammation, airspace hemorrhage, and airspace edema in LPS-challenged WT and LPS-challenged TTP^KO^ mice **(H)**. Statistical analysis in G and H was performed using Student’s *t*-test. *p < 0.05; **p < 0.01; ***p < 0.001; ****p < 0.0001.

In order to explore the role of TTP in ALI, TTP^KO^ and littermate control WT mice were subjected to ALI through oropharyngeal aspiration of endotoxin (LPS) (Single dose; 10 μg LPS/mouse) for a period of 72 h. While saline-treated TTP^KO^ and WT groups had comparable numbers of total immune cells in the BALF (saline-treated WT; 58 × 10^3^ ± 15 × 10^3^, saline-treated TTP^KO^, 74 × 10^3^ ± 19 × 10^3^), LPS administration resulted in increased infiltration of immune cells in both the TTP^KO^ and the WT groups. The total number of recovered immune cells in LPS-challenged TTP^KO^ mice (4867 × 10^3^ ± 1167 × 10^3^) were ∼ fourfold higher as compared to LPS-challenged WT (1184 × 10^3^ ± 467 × 10^3^) mice (Figure 1A). Increases in total cell counts in LPS-challenged TTP^KO^ mice were attributed to a significant increase in neutrophil (Figure 1B), macrophage (Figure 1C), and lymphocyte counts (Figure 1D). These increases were associated with an increased injury to the pulmonary vascular barrier, as depicted by the presence of red blood cells in the cytospins prepared from the BALF fluid of TTP^KO^ mice (Figure 1E; right panel, black arrow) versus control LPS-challenged WT mice (Figure 1E; left panel). Histologically, the lungs of LPS-challenged WT mice were characterized by mild to moderate consolidation (∼ 26% of total area of lung section), two- to fourfold increase in alveolar septal thickening (broken green arrow), moderate perivascular and airspace edema, and perivascular inflammation (Figures 1F–H). In contrast, the lung injury in LPS-challenged TTP^KO^ mice was characterized by severe consolidation (>90% of total area of lung section) (Figures 1F,G) that included infiltration of neutrophils, edema, fibrin, and airspace hemorrhage within the airway and alveolar lumen, multifocal loss of bronchiolar epithelium with infiltration of neutrophils and red blood cells within the bronchiolar lumen, and moderate to severe perivascular edema and inflammation (Figures 1F–H). Of note, ∼50% LPS-challenged TTP^KO^ mice succumbed to LPS challenge before 72-h and these had to be excluded from the analysis. These data suggest that systemic loss of TTP results in extreme susceptibility of mice to LPS-induced ALI.

### TTP Deletion in Myeloid Cells Results in Increased LPS-Induced ALI in Mice

**FIGURE 2:**
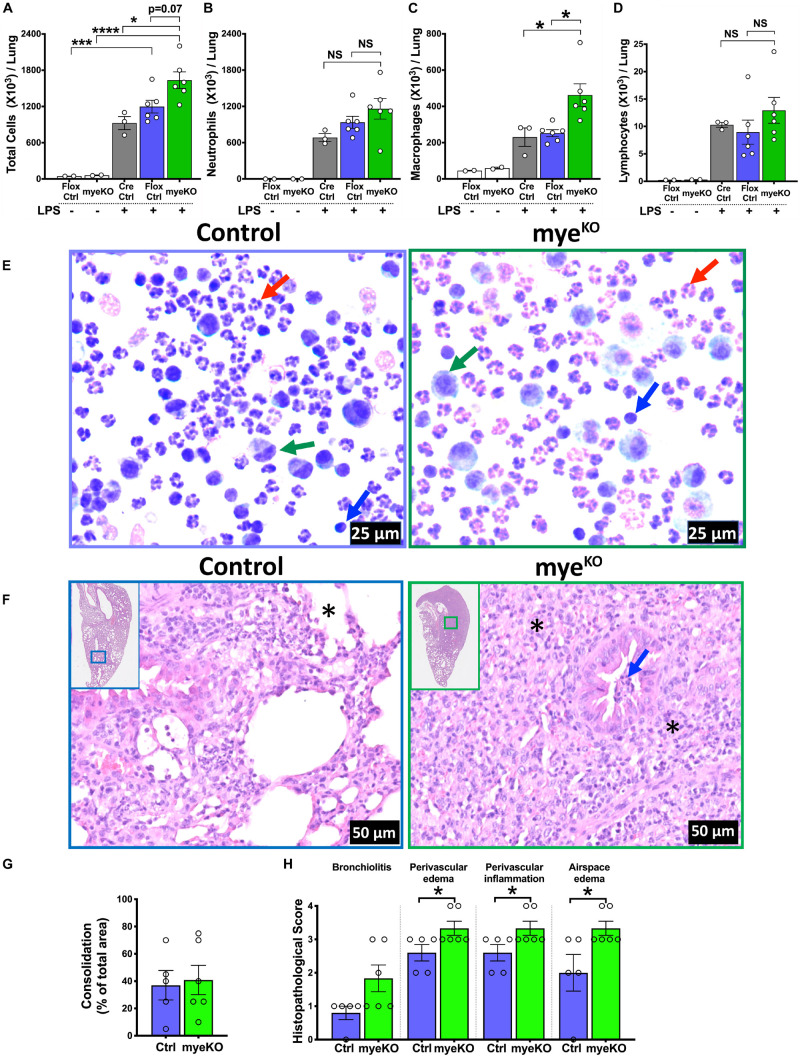
Myeloid-TTP deficiency exacerbates LPS-induced ALI in mice. Total cell counts **(A)** in the harvested BALF of adult saline- (white bars; *N* = 2 each for Flox control and TTP^myeKO^) or LPS-challenged Cre Control (Cre^+/+^/*Zfp36*^WT/WT^, gray bar; *N* = 3), Flox Control (Cre^– /–^ /*Zfp36*^Flox/Flox^, purple bar; *N* = 6), and TTP^myeKO^ (Cre^+/+^/*Zfp36*^Flox/Flox^, green bar; *N* = 6) mice. Differential cell counts are shown for neutrophils **(B)**, macrophages **(C)**, and lymphocytes **(D)**. Data are represented as mean ± SEM. Statistical analysis was performed by one-way ANOVA followed by Tukey’s correction for multiple comparisons. NS, non-significant; *p < 0.05; ***p < 0.001; ****p < 0.0001. Representative photomicrographs of Wright–Giemsa stained BALF cytospins from LPS-challenged Flox Control (**E**; Left) and TTP^myeKO^ (**E**; right) mice. Neutrophil (red arrow), macrophage (green arrow), lymphocyte (blue arrow) (original magnification ×400). Representative photomicrographs from H&E-stained left lung lobe sections from post-72 h LPS-challenged Flox Control (**F**; Left) and TTP^myeKO^ (**F**; right) mice (original magnification ×200). Asterisk represents alveolar spaces minimally obliterated in Flox control (**F**; left) and severely obliterated in LPS-challenged TTP^myeKO^ mice (**F**; right). Bronchiolar lumen neutrophilic infiltrates (blue arrow), absent in LPS-challenged Flox control (**F**; left) but present in LPS-challenged TTP^myeKO^ (**F**; right). Semiquantitative histopathological scoring for consolidation **(G)** is shown as a percent of total surface area of the lung section affected in LPS-challenged Flox control and LPS-challenged TTP^myeKO^ mice. Semiquantitative histopathological scoring of lung sections for bronchiolitis, perivascular edema, perivascular inflammation, and airspace edema in LPS-challenged Flox control and LPS-challenged TTP^myeKO^ mice **(H)**. *N* = 5 (Flox control); *N* = 6 (TTP^myeKO^). Statistical analysis in G and H was performed using Student’s *t*-test. *p < 0.05.

In order to explore the role of myeloid cell-specific TTP on inflammatory response in ALI, myeloid cell-specific TTP deficient mice (TTP^myeKO^; Cre^+/+^/*Zfp36*^Flox/Flox^) and control (Cre control; Cre^+/+^/*Zfp36*^WT/WT^ and Flox control; Cre^–/–^/*Zfp36*^Flox/Flox^) mice were challenged with LPS. Similar to saline-treated WT and TTP^KO^ groups, saline-treated control and TTP^myeKO^ groups had comparable numbers of total immune cells (Flox control; 46 × 10^3^ ± 1 × 10^3^, TTP^myeKO^; 60 × 10^3^ ± 5 × 10^3^). LPS administration resulted in increased numbers of immune cells in TTP^myeKO^ as well as both the control groups. This increase in total cell counts was, however, significantly greater in TTP^myeKO^ (1636 × 10^3^ ± 136 × 10^3^) compared to Cre control (925 × 10^3^ ± 109 × 10^3^) mice. The increase in cellular recovery did not reach statistical significance in LPS-challenged TTP^myeKO^ when compared to the LPS-challenged Flox control group (*p* = 0.07) (Figure 2A). This effect was comparable in both sexes (data not shown). Of note, the increase in cellular infiltration was ∼ threefold less in LPS-challenged TTP^myeKO^ (1636 × 10^3^ ± 136 × 10^3^) (Figure 2A) when compared to LPS-challenged TTP^KO^ mice (4867 × 10^3^ ± 1167 × 10^3^) (Figure 1A). While neutrophil counts were comparable between LPS-challenged TTP^myeKO^ and LPS-challenged control mice (Figures 2B,E), macrophage counts were significantly elevated in the BALF obtained from LPS-challenged TTP^myeKO^ mice compared to the two groups of control mice (Figures 2C,E). Lymphocyte counts did not differ between the LPS-challenged TTP^myeKO^ and the two groups of control mice (Figures 2D,E). Histopathological analysis revealed comparable levels of lung consolidation with widespread inflammatory cellular infiltrates within the airspaces of both LPS-challenged TTP^myeKO^ and Flox control mice; however, perivascular edema, perivascular inflammation, and airspace edema were somewhat exaggerated in LPS-challenged TTP^myeKO^ mice compared to the Flox control group (Figures 2F–H). Unlike LPS-challenged TTP^KO^ mice, airspace hemorrhage was not observed in any of the groups. All the LPS-challenged TTP^myeKO^ mice survived LPS challenge, as compared to the LPS-challenged TTP^KO^ mice, in which ∼50% mortality was observed. These data indicate that myeloid cell-specific TTP is essential for protection against ALI.

### Systemic TTP Overexpression (TTP^ΔARE^) Mitigates LPS-Induced ALI in Mice During Acute and Sub-Acute Course of Lung Injury

**FIGURE 3:**
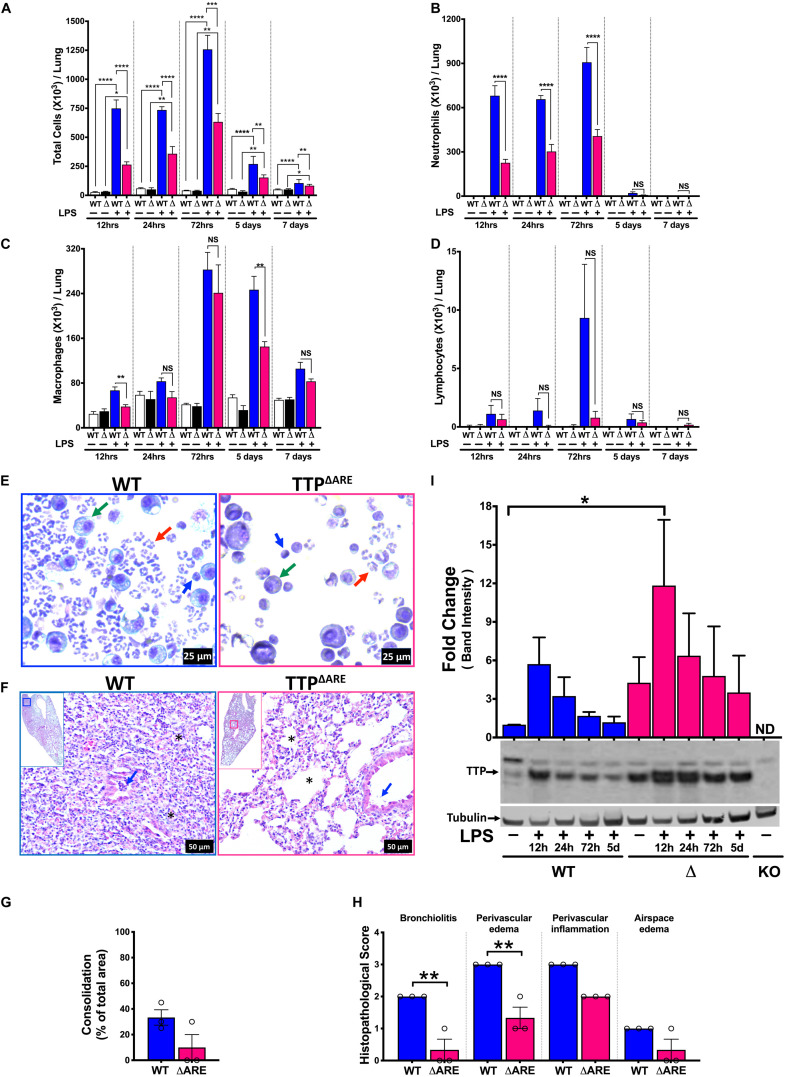
Systemic TTP overexpression (TTP^ΔARE^) mitigates LPS-induced ALI in mice during acute and sub-acute course of lung injury. Total cell counts **(A)** in the BALF from adult WT and TTP^ΔARE^ mice are shown at 12h, 24h, 72h, 5 days, and 7 days, respectively, post-LPS challenge. Differential cell counts are shown for neutrophils **(B)**, macrophages **(C)**, and lymphocytes **(D)** at various time points as above. Data are represented as mean ± SEM. Statistical analysis was performed by one-way ANOVA followed by Tukey’s correction for multiple comparisons in **A–D**. NS, non-significant; *p < 0.05, **p < 0.01, ***p < 0.001, ****p < 0.0001. *N* = 3–4 for saline control (WT and TTP^ΔARE^) mice at all time points; *N* = 6–8 for LPS-challenged mice (WT and TTP^ΔARE^) at all time points. Representative photomicrographs of Wright–Giemsa stained BALF cytospins of WT (**E**; left panel) and TTP^ΔARE^ (**E**; right panel) mice. Neutrophil (red arrow), macrophage (green arrow), lymphocyte (blue arrow) (original magnification ×400). Representative photomicrographs **(F)** from H&E-stained left lung lobe sections from LPS-challenged adult WT (**F**; left panel) and TTP^ΔARE^ (**F**; right panel) mice (original magnification ×200). Bronchiolar lumen neutrophilic infiltrates (blue arrow) and alveolar obliteration (*) present in the WT mice (**F**; left panel) but not in the TTP^ΔARE^ mice (**F**; right panel). Consolidation is shown as a percent of total surface area of the lung section affected **(G)** in WT and TTP^ΔARE^ mice. Histopathological scoring for bronchiolitis, perivascular edema, perivascular inflammation, and airspace edema is shown **(H)** in WT and TTP^ΔARE^ mice. *N* = 3 **(G,H)**. Statistical analysis in **G,H** was performed using Student’s *t*-test. **p < 0.01. A representative immunoblot and corresponding quantification of the immunoblots **(I)** showing TTP expression in WT (lanes 1–5: saline, LPS challenged lungs at 12h, 24h, 72h, and 5days, respectively) and TTP^ΔARE^ (lanes 6–10: saline, LPS challenged lungs at 12h, 24h, 72h, and 5days, respectively) mice lungs. The lane labeled as KO contains an equivalent amount of protein from the TTP^KO^ (negative control) mouse lung. Tubulin was used as a loading control. Statistical analysis was performed by one-way ANOVA. Data are represented as mean ± SEM. *N* = 3. *p < 0.05.

Next, we examined whether the systemically TTP overexpressing (TTP^ΔARE^) mice exhibit protection against ALI. In experimental ALI, while time points earlier than 72 h of LPS-challenge represent acute phases of ALI, later time points represent somewhat sub-acute to chronic or resolution phases of endotoxin-induced ALI in mice (22). Therefore, we examined both LPS-challenged WT and LPS-challenged TTP^ΔARE^ mice over time points representing acute to subacute phases, i.e., 12 h, 24 h, 72 h, 5 days, and 7 days. The numbers of inflammatory cells in BALF did not differ in saline-treated WT (26.6 × 10^3^ ± 5.4 × 10^3^, 59.3 × 10^3^ ± 6.2 × 10^3^, 41.8 × 10^3^ ± 2.1 × 10^3^, 54.1 × 10^3^ ± 5.0 × 10^3^, and 50 × 10^3^ ± 2.8 × 10^3^ cells at 12 h, 24 h, 72 h, 5 days, and 7 days, respectively) and saline-treated TTP^ΔARE^ mice (31.2 × 10^3^ ± 4.1 × 10^3^, 51.2 × 10^3^ ± 14.0 × 10^3^, 38.7 × 10^3^ ± 5.0 × 10^3^, 31.6 × 10^3^ ± 7.9 × 10^3^, and 50.8 × 10^3^ ± 3.6 × 10^3^ cells at 12 h, 24 h, 72 h, 5 days, and 7 days, respectively) (Figure 3A).

Lipopolysaccharide administration resulted in increased numbers of total cells in BALF from both WT and TTP^ΔARE^ mice when compared to saline administration (Figure 3A). Total cell counts in BALF from LPS-challenged WT mice were 748.9 × 10^3^ ± 72.7 × 10^3^, 735.6 × 10^3^ ± 28.0 × 10^3^, 1258 × 10^3^ ± 121 × 10^3^, 270 × 10^3^ ± 25.0 × 10^3^, and 115.4 × 10^3^ ± 6.9 × 10^3^ at 12 h, 24 h, 72 h, 5 days, and 7 days time points, respectively (Figure 3A). Interestingly, significantly reduced numbers of immune cells were recovered from the lungs of LPS-challenged TTP^ΔARE^ mice compared to lungs of LPS-challenged WT mice at all time points examined post LPS-challenge (264.6 × 10^3^ ± 24.0 × 10^3^, 358.4 × 10^3^ ± 62.1 × 10^3^, 633.1 × 10^3^ ± 71.6 × 10^3^, 153.3 × 10^3^ ± 9.4 × 10^3^, and 83.2 × 10^3^ ± 4.3 × 10^3^ at 12 h, 24 h, 72 h, 5 days, and 7 days, respectively) (Figure 3A).

The decrease in total cell counts in LPS-challenged TTP^ΔARE^ mice was contributed by significantly reduced numbers of neutrophils at 12, 24, and 72 h (Figures 3B,E). Interestingly, however, the total numbers of macrophages were only significantly different between LPS-challenged WT and LPS-challenged TTP^ΔARE^ mice at 12 h and 5 days time points (Figures 3C,E). Lymphocyte counts were not significantly different in LPS-challenged TTP^ΔARE^ mice compared to LPS-challenged WT mice (Figures 3D,E). Cellular counts followed the same trend in both the sexes (data not shown). Reduced cellular infiltration was also evident in cytological slides prepared from LPS-challenged WT and LPS-challenged TTP^ΔARE^ BAL fluid, which showed the sparsely present neutrophils, macrophages, and lymphocytes in LPS-challenged TTP^ΔARE^ mice compared to dense presence of these cells in LPS-challenged WT mice (Figure 3E). Histologically, LPS-challenged WT lungs exhibited ∼ 33% lung consolidation with significantly increased infiltration of immune cells within the airspaces, bronchiolitis, perivascular edema, and inflammation (Figures 3F–H). In contrast, bronchiolitis and perivascular edema were significantly attenuated in LPS-challenged TTP^ΔARE^ mice. Lung consolidation, perivascular inflammation, and airspace edema were not significantly different between the two groups, and airspace hemorrhage was not observed in any group (Figures 3F–H).

We next analyzed the changes in the protein expression levels of TTP over the course of ALI in WT and TTP^ΔARE^ mice whole lung tissue. As shown in Figure 3I, basal expression levels of TTP were higher in TTP^ΔARE^ versus WT lungs. Upon LPS administration, the expression levels of TTP increased in both WT and TTP^ΔARE^ mice lungs by 12 h post LPS administration. This increase was significant in TTP^ΔARE^ mice lungs as compared to unchallenged WT mice lungs. The levels of TTP started reducing at subsequent time points in both WT and TTP^ΔARE^ lungs. While the TTP expression decreased to basal levels in WT mice, the TTP expression remained at relatively higher levels in the TTP^ΔARE^ mice lungs at all subsequent time points. These data show that the presence of higher levels of TTP under basal conditions, combined with a significant increase at 12 h post-LPS challenge and maintenance of higher expression levels at later time points post LPS challenge, protects TTP^ΔARE^ mice from ALI.

### Protection From ALI in TTP Overexpressing Mice (TTP^ΔARE^) Is Associated With Reduced Secretion of Neutrophil Chemoattractants and Pro-inflammatory Cytokines

**FIGURE 4:**
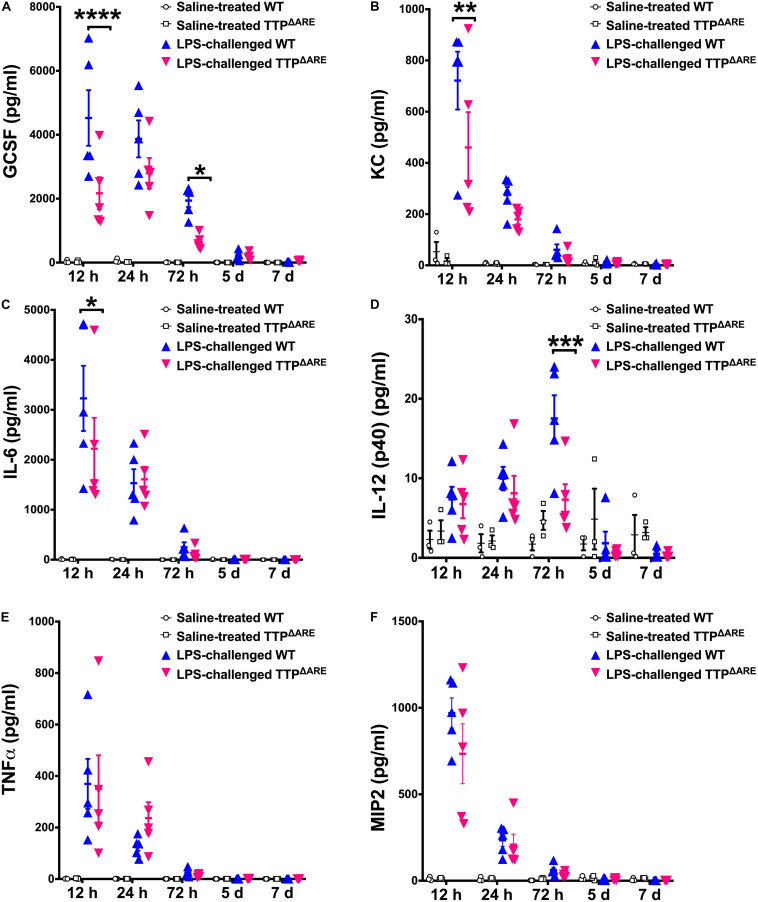
BALF levels of key pro-inflammatory cytokines and chemokines are significantly reduced in TTP overexpression mice compared to WT control mice. Levels of G-CSF **(A)**, KC **(B)**, IL-6 **(C)**, IL-12 (p40) **(D)**, TNFα **(E)**, and MIP2 **(F)** in the BALF from adult WT and TTP^ΔARE^ mice are shown. Saline-treated WT (open circles), saline-treated TTP^ΔARE^ (open squares), LPS-challenged WT (closed blue triangles), and LPS-challenged TTP^ΔARE^ (closed magenta triangles). Statistical analysis was performed by two-way ANOVA followed by Tukey’s correction for multiple comparisons. Data are represented as mean ± SEM. *N* = 3 (WT and TTP^ΔARE^ saline control); *N* = 5 (WT and TTP^ΔARE^ LPS). *p < 0.05, **p < 0.01, ***p < 0.001, ****p < 0.0001.

Cellular recruitment within the lung in response to LPS challenge is mediated by the production of chemoattractants. Therefore, next we sought to examine the levels of inflammatory cytokines and chemokines within the BALF of LPS-challenged WT and LPS-challenged TTP^ΔARE^ mice. Of the 25 cytokines/chemokines analyzed, four cytokines/chemokines, including G-CSF, KC, IL-6, and IL-12p40, were found to be significantly different between the LPS-challenged WT and the LPS-challenged TTP^ΔARE^ mice. G-CSF was significantly reduced in LPS-challenged TTP^ΔARE^ compared to LPS-challenged WT mice at 12 and 72 h; KC and IL-6 were significantly reduced at 12 h; and IL-12 (p40) was significantly reduced at 72 h post-LPS challenge (Figures 4A–D). Interestingly, the levels of TNFα (Figure 4E) and MIP2 (Figure 4F), two known TTP targets, were not significantly different between the two groups.

### Non-hematopoietic Cell-Specific TTP Overexpression Within the Lung Is Essential for Protection Against ALI

To determine the cell-specific role of TTP levels in ALI, we modulated TTP levels in hematopoietic progenitor cells (HPCs) and non-HPCs. In order to test whether donor HPCs repopulate the recipient mouse lungs, we first made bone marrow chimeras in which total body irradiated WT mice were transplanted with HPCs from a mouse expressing green fluorescent protein (GFP) in their somatic cells. We found that greater than 90% of the cells recovered from the BALF of these mice were GFP positive (data not shown).

**FIGURE 5:**
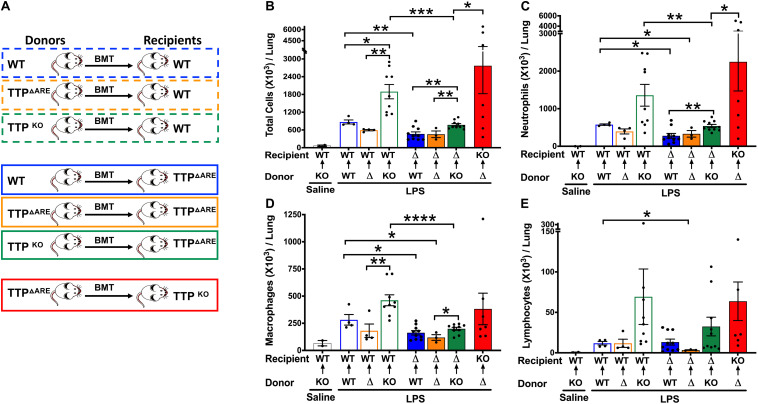
Effect on ALI following bone marrow irradiation and reconstitution of WT, TTP overexpressing (TTP^ΔARE^), and TTP knockout (TTP^KO^) mice. Experimental design **(A)** for bone marrow irradiation and reconstitution experiment followed by LPS-induced ALI. Total cell counts **(B)** in the BALF from adult recipient WT, TTP^ΔARE^, and TTP^KO^ mice post 72 h after LPS-challenge. Open black bar (saline administered recipient WT mice); open blue bar (WT→WT), open orange bar (TTP^ΔARE^→WT), open green bar (TTP^KO^→WT), solid blue bar (WT→TTP^ΔARE^), solid orange bar (TTP^ΔARE^→TTP^ΔARE^), solid green bar (TTP^KO^→TTP^ΔARE^), solid red bar (TTP^ΔARE^→TTP^KO^). Differential cell counts are shown for neutrophils **(C)**, macrophages **(D)**, and lymphocytes **(E)**. Statistical analysis was performed by one-way ANOVA followed by Tukey’s correction for multiple comparisons within the three recipient groups and Student’s *t*-test between the three recipient groups. Data are represented as mean ± SEM. *N* = 2 (saline control); *N* = 4 (WT→WT), *N* = 4 (TTP^ΔARE^→WT); *N* = 9 (TTP^KO^→WT), *N* = 10 (WT→TTP^ΔARE^), *N* = 3 (TTP^ΔARE^→TTP^ΔARE^), *N* = 11 (TTP^KO^→TTP^ΔARE^), *N* = 7 (TTP^ΔARE^→TTP^KO^). *p < 0.05, **p < 0.01, ***p < 0.001, ****p < 0.0001.

Next, we generated three bone marrow chimeras in which irradiated WT recipient mice received HPCs from either WT (WT→WT), TTP^ΔARE^ (TTP^ΔARE^→WT), or TTP^KO^ (TTP^KO^→WT) mice (Figure 5A). While saline-treated TTP^KO^→WT chimeras had no signs of cellular infiltration that included total cells (Figure 5B), neutrophils (Figure 5C), macrophages (Figure 5D), and lymphocytes (Figure 5E), LPS-challenged WT→WT, TTP^ΔARE^→WT, and TTP^KO^→WT chimera mice had significant cellular recruitment, as indicated by BALF total cellular counts (Figure 5B), neutrophils (Figure 5C), and macrophages (Figure 5D). While LPS-challenged TTP^ΔARE^→WT mice had somewhat reduced cellular infiltration as compared to LPS-challenged WT→WT chimeras (Figures 5B–D), the LPS-challenged TTP^KO^→WT chimera mice had remarkably higher number of BAL cellular counts (Figures 5B–D). Lymphocyte counts did not differ significantly between the three groups (Figure 5E).

**FIGURE 6:**
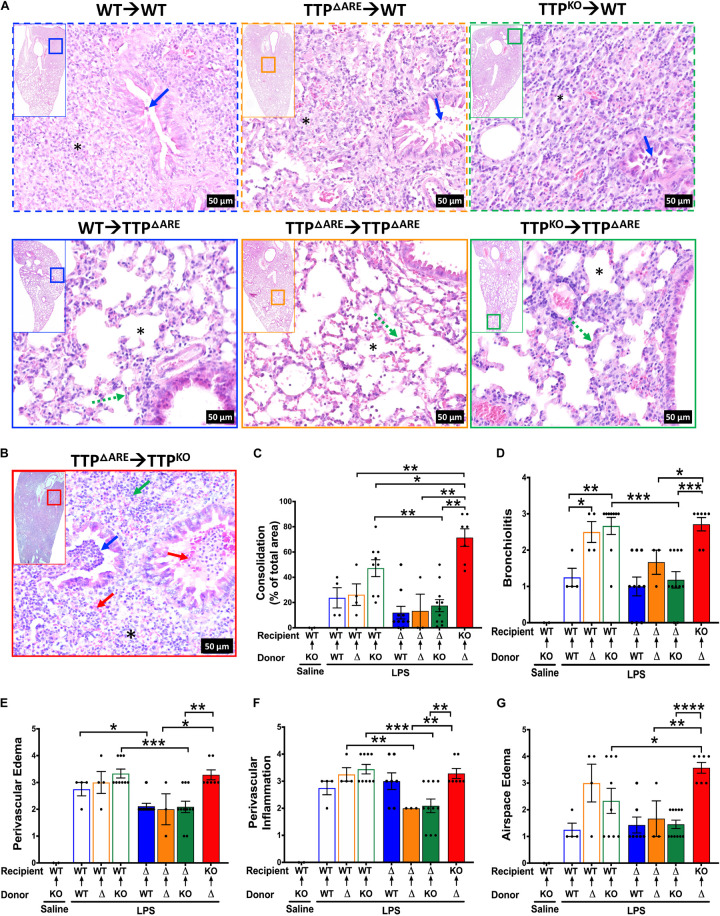
Histopathological assessment of ALI following bone marrow irradiation and reconstitution of WT, TTP overexpression (TTP^ΔARE^), and TTP knockout (TTP^KO^) mice. Representative photomicrographs from H&E-stained left lung lobe sections from LPS-challenged recipient WT (**A**; top panel), LPS-challenged recipient TTP^ΔARE^ (**A**; bottom panel), and LPS-challenged recipient TTP^KO^ **(B)** mice (original magnification ×200). Bronchiolar lumen neutrophilic infiltrates (blue arrow), presence (* in obliterated alveolar space) or absence (* in open alveolar space) of alveolar obliteration, alveolar septal thickening (green dotted line), bronchiolar and alveolar space hemorrhage (red arrow), alveolar space neutrophils (green arrow). Consolidation is shown as a percent of total surface area of the lung section affected **(C)** in all chimeras. Histopathological scoring for bronchiolitis **(D)**, perivascular edema **(E)**, perivascular inflammation **(F)**, and airspace edema **(G)** are shown in all chimeras. WT recipient (open blue, orange, green bars); TTP^ΔARE^ recipient (solid blue, orange, green bars); TTP^KO^ recipient (solid red bar). *N* = 4 (WT→WT), *N* = 4 (TTP^ΔARE^→WT), *N* = 9 (TTP^KO^→WT), *N* = 10 (WT→TTP^ΔARE^), *N* = 3 (TTP^ΔARE^→TTP^ΔARE^), *N* = 11 (TTP^KO^→TTP^ΔARE^), *N* = 7 (TTP^ΔARE^→TTP^KO^). Data are represented as mean ± SEM. Statistical analysis in **C–G** was performed by one-way ANOVA followed by Tukey’s correction for multiple comparisons within the three recipient groups and Student’s *t*-test between the three recipient groups. *p < 0.05; **p < 0.01; ***p < 0.001; ****p < 0.0001.

Histologically, ∼23, 26, and 47% of the lung parenchyma was consolidated in LPS-challenged WT→WT, LPS-challenged TTP^ΔARE^→WT, and LPS-challenged TTP^KO^→WT group, respectively (Figures 6A top panel, 6C). Consolidated parenchyma was characterized by the presence of large infiltrates of neutrophils and macrophages within the airspaces (alveolar and airway). Other histological findings included the presence of edema and immune cells within the perivascular spaces, bronchial lumen cellular infiltrates, airspace edema, and occasional bronchoalveolar lymphoid aggregates (Figures 6D–G). These data suggest that while baseline expression of TTP in HPCs is essential for protection against exaggerated ALI, its overexpression in these cells does not confer significant additional advantage.

Next, we generated bone marrow chimeras in which irradiated TTP^ΔARE^ recipient mice received HPCs from either WT (WT→TTP^ΔARE^), TTP^ΔARE^ (TTP^ΔARE^→TTP^ΔARE^), and TTP^KO^ (TTP^KO^→TTP^ΔARE^) mice (Figure 5A). As compared to WT recipient chimera counterparts, LPS-challenged WT→TTP^ΔARE^, LPS-challenged TTP^ΔARE^→TTP^ΔARE^, and LPS-challenged TTP^KO^→TTP^ΔARE^ chimeras had significantly lower degrees of cellular recruitment, that included total cells (Figure 5B, blue solid bar, orange solid bar, green solid bar), neutrophils (Figure 5C, blue solid bar, orange solid bar, green solid bar), and macrophages (Figure 5D, blue solid bar, orange solid bar, green solid bar). Lymphocyte counts were significantly reduced in LPS-challenged TTP^ΔARE^→TTP^ΔARE^ compared to the LPS-challenged WT→WT chimeras (Figure 5E). Although the LPS-challenged TTP^KO^→TTP^ΔARE^ chimeras had significantly higher cellular recruitment as compared to LPS-challenged WT→TTP^ΔARE^ and LPS-challenged TTP^ΔARE^→TTP^ΔARE^ chimeras, the extent of recruitment was much diminished in LPS-challenged TTP^KO^→TTP^ΔARE^ chimera as compared to LPS-challenged TTP^KO^→WT chimera (Figure 5B).

Histologically, ∼11, 13%, 17% of the lung parenchyma was consolidated in LPS-challenged WT→TTP^ΔARE^, LPS-challenged TTP^ΔARE^→TTP^ΔARE^, and LPS-challenged TTP^KO^→TTP^ΔARE^, respectively (Figures 6A bottom panel, Figure 6C). Histologically, mild increases in septal thickening with cellular infiltration, mild bronchiolitis, perivascular edema, inflammation, and airspace edema, and occasional BALTs were evident in all the three groups (Figures 6D–G, solid blue, orange, and green bars). These data suggest that enhanced expression of TTP in lung non-HPC populations conferred significant protection against ALI. Further, this protection is somewhat compromised in the absence of baseline levels of TTP in HPCs. A tabular summary of differential cellular and ALI responses in various chimeric mice is included in Supplementary Table 1.

Finally, we generated bone marrow chimeras in which irradiated TTP^KO^ recipient mice received HPCs from TTP^ΔARE^ (TTP^ΔARE^→TTP^KO^). As expected, the cellular counts were significantly higher than any of the other LPS-challenged chimeras (Figures 5B–E, Red solid bar). However, total cellular counts in LPS-challenged TTP^ΔARE^→TTP^KO^ chimera were ∼ twofold lower than the LPS-challenged TTP^KO^ mice (Figure 1). Additionally, none of the LPS-challenged TTP^ΔARE^→TTP^KO^ chimeras succumbed to ALI, as compared to 50% mortality in LPS-challenged TTP^KO^ mice (Figure 1). These data suggest that complete loss of TTP in lung non-HPC populations significantly exaggerates ALI, and that overexpression of TTP in HPCs may provide partial protection in severe ALI. Histologically, this group exhibited the most severe lung injury, characterized by ∼70% lung consolidation with neutrophils, macrophages, fibrin, and edema, severe bronchiolitis, and moderate to severe bronchiolar and alveolar hemorrhages (Figures 6B–G).

## Discussion

Tristetraprolin knockout (TTP^KO^) mice exhibit a systemic inflammatory syndrome that is characterized by cachexia, polyarticular synovial arthritis, dermatitis, and myeloid hyperplasia (4). However, the lungs of TTP^KO^ mice exhibit very little spontaneous inflammation, characterized by the presence of rare foci of leucocytic infiltrates within the pulmonary parenchyma (8). These rare leucocytic infiltrates are abrogated upon combined deletion of TTP and TNF receptors, indicating the role for TNF activity in leucocytic infiltration (8). Myeloid cell-specific loss of TTP (TTP^myeKO^) does not recapitulate the TTP^KO^ phenotype, indicating that non-myeloid cells are required for the overall manifestation of the TTP deficiency syndrome (16). However, TTP^myeKO^ mice were found to be hypersensitive to endotoxin-induced systemic inflammation, particularly through exaggerated TNF production, delineating the critical role of myeloid cell-specific TTP in protection against systemic injury and inflammation (16). The role of TTP in localized lung inflammation, however, has remained unexplored. Therefore, in this study we sought to explore the role of TTP in ALI. We hypothesized that TTP modulates acute lung inflammation and that cell-specific modulation of TTP levels will differentially affect the outcome of acute lung inflammation.

The unchallenged TTP^KO^ mice display minor degrees of leukocytic infiltration in lung parenchyma that were thought to be contributed by TNF activity (8). Here, in LPS-challenged TTP^KO^ mice, immune cell infiltration was ∼ fourfold higher as compared to LPS-challenged WT mice. In fact, the susceptibility of TTP^KO^ mice to LPS-induced ALI was so severe that ∼50% TTP^KO^ mice succumbed to LPS-challenge within 24–72 h post-LPS administration, while no mortality was observed in LPS-challenged WT mice. The surviving TTP^KO^ mice displayed severe pulmonary pathology with exaggerated airspace and interstitial neutrophilic infiltration, exaggerated edema, vascular congestion, and lung injury that included epithelial and endothelial damage. It is likely that the increased production of inflammatory mediators, that are otherwise regulated by TTP, in LPS-challenged TTP^KO^ mice, contribute to their worsened pulmonary pathology. One such mediator, TNF, is already established to be highly secreted in TTP^KO^ mice (4, 6).

Macrophages are the primary source of TNF in ALI (23, 24) although alveolar epithelial cells have also been suggested to release TNF in LPS-induced lung inflammation (25). Accordingly, we reasoned that, if macrophages are the primary source of TNF, deletion of TTP in myeloid cells would enhance its activity, leading to worsening of pulmonary pathology. Although the LPS-challenged TTP^myeKO^ mice had exaggerated pulmonary pathology as compared to LPS-challenged WT mice, the extent of tissue damage and neutrophilic infiltration was not as severe as in LPS-challenged TTP^KO^ mice. These differences in the sensitivity of systemic versus myeloid cell-specific TTP-deficient mice indicate that TTP in cells other than myeloid-cells may play critical roles in modulating endotoxin-induced ALI. These data suggest that while loss of myeloid cell-specific TTP worsens the ALI, TTP expression in non-myeloid cells confers significant protection.

Clinically, the numbers of neutrophils within the BALF of patients with ARDS have been shown to correlate with the severity of disease and poor outcome (26, 27). Despite being the first line of defense against pathogenic insults, excessive recruitment and activation of neutrophils leads to lung tissue damage and loss of lung function (28, 29). Therefore, targeting neutrophilic recruitment through suppressed release of key neutrophil chemoattractants may be an attractive therapeutic strategy against ALI. We observed correlations between TTP deficiency or TTP overexpression and neutrophilic inflammation. For example, BALF counts and tissue infiltration of neutrophils were overwhelming in the LPS-challenged TTP^KO^ mice. These outcomes were also exaggerated, but to a lesser degree, in LPS-challenged TTP^myeKO^ mice. On the other hand, these outcomes were significantly attenuated in the LPS-challenged TTP^ΔARE^ mice. Our findings are in line with previous reports in other tissues. For instance, massive infiltration of neutrophils has been shown to occur in the skin of TTP^KO^ mice subjected to psoriasis-like inflammation (30), whereas reduced airway neutrophilic infiltration was shown in mice genetically modified to express constitutively active endogenous unphosphorylated TTP following challenge with cigarette smoke (31).

A number of studies have shown that TTP is phosphorylated at multiple sites by p38-regulated kinase MAPK-activated protein kinase 2 (MAPKAPK2) and that TTP activity is significantly affected by its phosphorylation status (32–34). TTP phosphorylation has been shown to result in reduced TTP activity/reduced binding of TTP to AREs, thus resulting in stabilization of its target mRNAs (35). Regulation of TTP activity by p38 MAPK was in fact shown to result in a biphasic response of TNFα-induced IL-6 expression in human bronchial smooth muscle cells (36). Conversely, dephosphorylation of TTP resulted in reduced expression of IL-6 and IL-8 in A549 lung epithelial cells (37). Since LPS is an inducer of both TTP and p38 MAPK, TTP would be expected to undergo phosphorylation and inactivation shortly after LPS challenge. However, the effect of TTP phosphorylation is transient and would be expected to be reversed upon dephosphorylation when p38 is turned off. Although we did not track the phosphorylation status of TTP in various lung cells during ALI, we speculate that the P38-mediated phosphorylation of TTP occurs well before 12h post LPS challenge and is reversed by 12 h time point. Consistent with our speculation, we found differential effect of TTP overexpression on late phase cytokines (KC) versus early phase cytokines (TNF) (Figure 4).

Tristetraprolin is a known regulator of key neutrophil chemoattractants including CXCL1/KC (8, 9) and CXCL2/MIP2 (8, 38). CXCL1/KC is a central chemokine in neutrophil recruitment into the airspace in ARDS (39–41). Clinically, increased concentrations of IL-8 (CXCL1/KC homolog in human), disease severity, and neutrophil migration into airspaces have been shown to be correlated (42–44). *Cxcl1* mRNA 3′UTR contains TTP-binding sites and has been previously demonstrated to be a direct target of TTP (8, 9). Consistent with this, the LPS-challenged TTP^ΔARE^ mice had significantly lower CXCL1/KC concentrations. In contrast, the two well characterized TTP targets, TNF and MIP2/CXCL2, were not significantly different between LPS-challenged WT and LPS-challenged TTP^ΔARE^ mice. These observations are in line with our previous report, in which no differences were observed in the levels of TNF and MIP2/CXCL2 in the serum of WT and TTP^ΔARE^ mice systemically challenged with LPS (15). These two inflammatory mediators peak early (before 6 h in the serum) (15), and we speculate that overexpressed TTP may be less effective at mRNA decay due to its phosphorylation status at these early time points.

G-CSF, another neutrophil chemoattractant, is also consistently detected within the BALF of ALI and ARDS patients and has been suggested to be associated with the accumulation and activation of neutrophils in ARDS (45). Plasma G-CSF levels have also been shown to correlate with clinical outcome in patients with ALI (27, 46). G-CSF has also been shown to exacerbate bleomycin induced lung injury in rats through marked infiltration of activating neutrophils (47). G-CSF levels have been found to be increased in TTP^KO^ mouse plasma (48). Although G-CSF has not been shown to be a direct TTP target, G-CSF mRNA 3′UTR in mouse possesses two TTP binding sites, UAUUUAU. The BALF G-CSF levels were significantly reduced in LPS-challenged TTP^ΔARE^ mice. G-CSF levels were also found to be significantly reduced in TTP^ΔARE^ mice in a previous study where we showed TTP^ΔARE^ mice to be significantly protected from exhibiting collagen-antibody induced arthritis (15).

Lipopolysaccharide-challenged TTP^myeKO^ mice exhibited milder ALI as compared to LPS-challenged TTP^KO^ mice, indicating that total loss of TTP expression in non-myeloid as well as myeloid cells contributes to severe ALI. On the other hand, systemic TTP overexpression conferred significant protection against LPS-induced ALI. Here, we addressed two logical questions: (1) whether enhanced levels of TTP in non-HPCs would ameliorate endotoxin-induced ALI, (2) whether enhanced levels of TTP in HPCs would confer protection against endotoxin-induced ALI. Towards this, employing various bone marrow chimeras, we investigated cell-specific roles of TTP in protection against ALI. In these experiments, we modulated TTP levels in HPCs by using bone marrow donors that were either WT, TTP^ΔARE^, or TTP^KO^. To modulate TTP levels in non-HPCs, WT, TTP^ΔARE^, or TTP^KO^ recipients were used. One limitation of this study was that since HPCs also include various non-myeloid populations, we were not able to specifically investigate the effect of TTP modulation in myeloid cells.

The bone marrow irradiation and reconstitution experiments revealed somewhat unexpected but interesting findings. As compared to WT→WT chimera, the ALI in TTP^ΔARE^→WT chimeras was somewhat attenuated, whereas the ALI in TTP^KO^→WT chimeras had worsened significantly. These data suggest that, when the TTP expression in non-HPCs is genetically unaltered (WT TTP in the recipient’s non-HPCs), the TTP overexpression in HPCs is partially protective against LPS-induced ALI. However, when the TTP expression was ablated in non-HPCs [TTP depleted in the recipient’s non-HPCs (TTP^ΔARE^→TTP^KO^ chimera)], the overexpression of TTP in the HPCs was not sufficient to confer protection against ALI. As compared to TTP^ΔARE^→TTP^KO^ chimeras, in TTP^KO^→TTP^ΔARE^ chimeras, the mere overexpression of TTP in non-HPCs (TTP overexpression in the recipient’s non-HPCs) provided significant protection, even though the HPCs were TTP-deficient. However, this protection was further enhanced when the normal levels of TTP were restored in the HPCs (WT→TTP^ΔARE^ chimera). Based on these data, we conclude here that the TTP levels in non-HPCs are critical in protection against ALI. Further, while the WT levels of TTP in HPCs are essential for additional protection, the overexpression of TTP in HPCs is not significantly advantageous. Since the non-HPCs population in lungs consists of a multitude of cell types including epithelial cells, endothelial cells, and fibroblasts, the cell-specific role of TTP still remains elusive.

Contribution of non-HPC-specific TTP toward modulation of inflammatory responses has also been suggested in previous reports. For instance, TTP regulation of TNF in keratinocytes, but not in myeloid or dendritic cells, was shown to protect mice from exacerbation of psoriasis-like pathology, development of spontaneous systemic inflammation, and dactylitis (30). Another study suggested a role of intestinal epithelial cell-specific TTP in exacerbation of acute colitis (49). Along similar lines, TTP depletion in myeloid cells did not replicate the TTP-deficiency phenotype (16), and the spontaneous reactive granulopoiesis seen in TTP^KO^ mice was suggested to be caused by a non-cell autonomous mechanism likely contributed by liver cells (48). All these studies indicated an essential role of TTP in non-HPCs in regulating inflammation. Among various non-HPC cell types in the lung, alveolar epithelial cells produce pro-inflammatory cytokines and chemokines (50). In *in vitro* conditions, LPS-challenged pulmonary type II epithelial cells have been shown to produce greater levels of neutrophil chemoattractants [IL-8, a human homolog for CXCL1 (KC), CXCL2 (MIP2), and CXCL5 (LIX)] indicating that TTP in lung alveolar epithelial cells may play an important role in regulating ALI (50). Consistent with these reports, while our data indicate critical roles of non-HPCs in lungs in mediating neutrophil recruitment to the airspaces, the exact identity of these non-HPCs remain unknown.

## Conclusion

**FIGURE 7:**
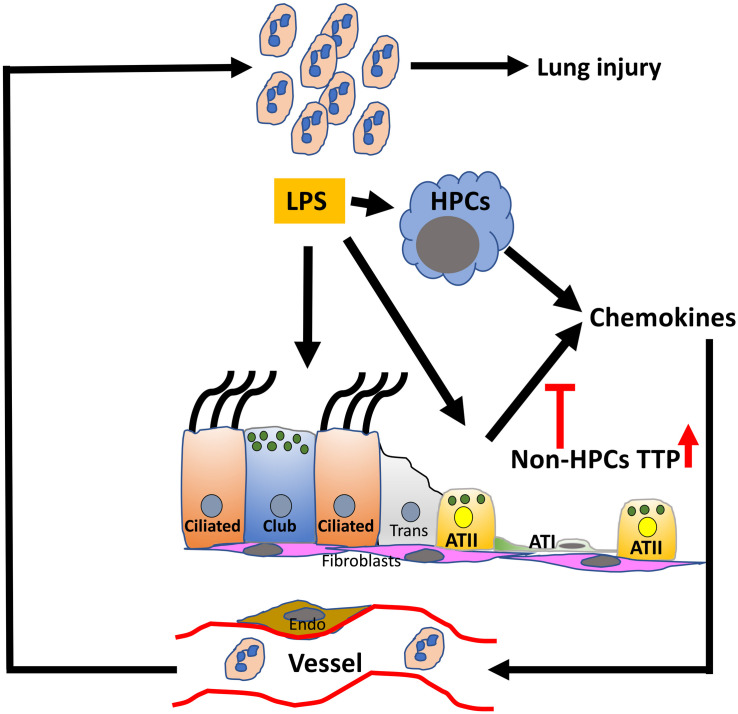
Conceptual model depicting the role of TTP in ALI. The resident cells, myeloid and epithelial, in the lung airspaces respond to the exposure of endotoxin, i.e., LPS. LPS-induced synthesis of chemokine production in the resident cells is modulated via TTP-dependent post-transcriptional regulation in a cell-specific manner. The current study suggests that enhanced TTP expression in non-hematopoietic progenitor cells (HPCs) suppresses the release of neutrophil chemoattractants and protects against ALI. Non-HPC populations include airway (Ciliated, Club, subepithelial cells) and alveolar type I and II cells (ATI and ATII), endothelial cells (Endo), and fibroblasts (fibro); TTP, tristetraprolin; LPS, lipopolysaccharide.

In conclusion, our results show that (a) enhancing the levels of TTP is protective against endotoxin-induced ALI; (b) the protective effect seen in TTP^ΔARE^ mice is attributable to reduced neutrophilic recruitment and, in turn, reduced lung damage; (c) reduced neutrophilic recruitment is attributed to reduced secretion of chemoattractants, particularly KC and G-CSF; and that, (d) TTP in non-HPCs plays an essential role in protection against endotoxin-induced ALI. Based on these results, we propose a model in which endotoxin damages epithelial cells within the lung, which then initiates a cascade of events leading to exaggerated release of proinflammatory mediators and neutrophilic chemoattractants, resulting in further lung damage and neutrophilic infiltration (Figure 7). In this process, TTP acts as an intracellular regulator for the expression of proinflammatory mediators and neutrophilic chemoattractants. TTP expression in the non-HPCs, i.e., most likely epithelial and endothelial cells, confers protection against endotoxin-induced ALI via suppression of mRNAs encoding proinflammatory mediators and neutrophilic chemoattractants. Hence, strategies to increase TTP expression or activity in non-HPCs together with HPCs population may prove beneficial for patients with ALI/ARDS. In future studies, TTP expression within the lung could also be investigated as a prognostic indicator for the severity of ALI/ARDS. Our studies could also have implications for the lung hyper-inflammation and potentially life-threatening cytokine storms in the severe coronavirus disease (COVID-19).

## Data Availability Statement

The raw data supporting the conclusions of this article will be made available by the authors, without undue reservation.

## Ethics Statement

The animal study was reviewed and approved by LSU IACUC.

## Author Contributions

SP conceived and designed the study, maintained the animal colony, and performed histopathological analyses. IC, TV, and BL conducted animal necropsies. YS, CB, and RL performed BALF cellularity assays. RL performed the intravenous injections. YS, TV, IC, and CB performed cytokine assays. JL performed the irradiations. SJ provided technical expertise on bone marrow transplantations and reviewed the manuscript. PB provided various transgenic and knockout mice and edited the manuscript. SP and YS wrote and reviewed the manuscript for intellectual contents. All authors contributed to the article and approved the submitted version.

## Conflict of Interest

The authors declare that the research was conducted in the absence of any commercial or financial relationships that could be construed as a potential conflict of interest.
