# Supplementary material for: Tristetraprolin Overexpression in Non-hematopoietic Cells Protects Against Acute Lung Injury in Mice
Source: Front Immunol. 2020 Sep 2;11:2164. doi: 10.3389/fimmu.2020.02164 (PMC7493631; doi:10.3389/fimmu.2020.02164)
Supplement: Supplementary file 1 [file Table_1.DOCX]

| **Chimera Characteristics** | **Inflammatory Immune Cells** | **Acute Lung Injury** |
| --- | --- | --- |
| Normal TTP Expression in all cells | Baseline | Baseline |
| TTP overexpression in HPCs | Baseline | Baseline |
| TTP depletion in HPCs | Exaggerated (++) | Exaggerated (++) |
| TTP overexpression in Non-HPCs | Reduced | Reduced |
| TTP overexpression in both non-HPCs and HPCs | Reduced | Reduced |
| TTP overexpression in non-HPCs but deletion in HPCs | Exaggerated (+) | Exaggerated (+) |
| TTP deletion in non-HPCs but overexpression in HPCs | Exaggerated (+++) | Exaggerated (+++) |

**Supplemental Table 1**
